# Sedation Mode During Endovascular Stroke Treatment in the Posterior Circulation—Is Conscious Sedation for Eligible Patients Feasible?

**DOI:** 10.3389/fneur.2021.711558

**Published:** 2021-09-17

**Authors:** Charlotte Sabine Weyland, Min Chen, Arne Potreck, Laura Bettina Jäger, Fatih Seker, Silvia Schönenberger, Martin Bendszus, Markus Möhlenbruch

**Affiliations:** Neurologische Klinik, UniversitätsKlinikum Heidelberg, Heidelberg, Germany

**Keywords:** endovascular stroke therapy, mechanical thrombectomy, posterior circulation, anesthesia management, conscious sedation, acute ischemic stroke

## Abstract

**Background and Purpose:** To compare safety and efficacy of conscious sedation (CS) vs. general anesthesia (GA) in endovascular stroke treatment (EST) of the posterior circulation (PC).

**Methods:** Retrospective single-center analysis of patients receiving EST for large-vessel occlusion (LVO) in PC between January 2015 and November 2020. Exclusion criteria were severe stroke syndromes (NIHSS > 20), decreased level of consciousness, intubation for transport, and second stroke within 3 months of follow-up. The primary endpoint was a favorable clinical outcome 90 days after stroke onset (mRS 0–2 or 3 if pre-stroke mRS 3). Secondary endpoints were the rate of EST failure and procedural complications.

**Results:** Of 111 included patients, 45/111 patients (40.5%) were treated under CS and 60/111 (54.0%) under GA. In 6/111 cases (5.4%), sedation mode was changed from CS to GA during EST. Patients treated under CS showed a lower mRS 90 days after stroke onset [mRS, median (IQR): 2.5 (1–4) CS vs. 3 (2–6) GA, *p* = 0.036] and a comparable rate of good outcome [good outcome, *n* (%): 19 (42.2) CS vs. 15 (32.6) GA, *p* = 0.311]. There was no difference in complication rates during EST (6.7% CS vs. 8.3% GA) or intracranial bleeding in follow-up imaging [*n* (%): 4 (8.9) CS vs. 7 (11.7) GA), *p* = 0.705]. The rate of successful target vessel recanalization did not differ (84.4% CS vs. 85.0 % GA).

**Conclusions:** In this retrospective study, EST of the posterior circulation under conscious sedation was for eligible patients comparably safe and effective to patients treated under general anesthesia.

## Key Points

- Endovascular Stroke Treatment for patients with ischemic stroke in the posterior circulation is safe and effective when compared to treatment under general anesthesia with a low rate of failed recanalization and complications.
- Eligible patients show a comparable rate of good functional outcome 90 days after stroke onset.

## Introduction

The mode of sedation during endovascular stroke treatment (EST) is a matter of debate in recent years, mainly regarding EST for large-vessel occlusions (LVO) in the anterior circulation. While conscious sedation (CS) can be time-saving and allows a faster treatment process between imaging and groin puncture, general anesthesia (GA) guarantees immobility of the patient, airway protection, and safe ventilation. Also, uncontrolled blood pressure during the procedure can have an impact on patient outcome. During GA blood pressure tends to be overall lower but is more controllable (1).

In patients with LVO of the anterior circulation, three randomized-controlled trials showed an equal clinical outcome for patients treated under CS or GA for LVO in the anterior circulation (2–4). More recently, a meta-analysis of the three studies mentioned above indicated that patients treated under GA may even have a better clinical outcome (5).

In comparison with the high-level evidence for the benefit of EST in the anterior circulation, there is a lack of evidence for treating patients with ischemic stroke and LVO of the posterior circulation. The BEST trial showed a higher rate of favorable outcome for patients with vertebrobasilar occlusions treated with EST compared to best medical treatment (BMT), but the high crossover rate after randomization represents a major limitation of the trial (6). In the Basilar Artery International Cooperation (BASIC) study (7), which randomized 300 patients until December 2019, EST failed to outperform BMT alone. In this underpowered study, the estimated risk reduction of mortality (16%) was not reached in the intervention arm. However, the intervention arm showed a risk reduction of 6.5% (44.2% compared to 37.7%). Subgroup analyses showed a favorable outcome for patients older than 70 years treated with EST and for patients with bridging i.v. thrombolysis before EST. After all, it remains uncertain if a convincing randomized trial for evidence of EST in the posterior circulation can be conducted. Indirect evidence for the benefit of EST in the posterior circulation remains, like the very high mortality rate, when the occlusion remains despite treatment (8). There is also indirect evidence from comparing outcome parameters and technical success rates with EST of the anterior circulation, where EST of the posterior circulation seems equally safe and effective (9, 10).

When it comes to sedation mode during EST of the posterior circulation contrarily to the anterior circulation, for EST in the posterior circulation, endotracheal intubation and treatment under GA are often deemed mandatory (11). Vertebrobasilar occlusion impairs the brainstem's blood perfusion resulting in disorientation, decreased level of consciousness, and loss of protective reflexes. Thus, patients with acute vertebrobasilar artery occlusions are often comatose on presentation and need to be intubated. Also, with loss of protective reflexes there is often a higher risk of aspiration and apnea requiring airway protection (12). However, some patients with LVO of the posterior circulation present with mild to moderate stroke syndromes (NIHSS 5–20) and clinically stable enough for EST under CS. Opposed to the broadly investigated sedation mode of anterior circulation stroke patients, it remains unclear if treating these patients under CS is comparably safe and effective.

The aim of this study was to compare eligible patients with acute ischemic stroke of the posterior circulation treated with state-of-the-art EST under CS compared to treatment under GA.

## Methods

For this study, a retrospective single-center analysis of an institutional review board–approved stroke database was performed. The tertiary stroke center's database with prospectively obtained patients was searched for patients receiving EST for LVO in the posterior circulation under CS between January 2015 and November 2020.

### Study Endpoints, Patient Selection, and Study Groups

The primary endpoint of this study was a favorable clinical outcome 90 days after stroke onset (mRS 0–2 or 3 if pre-stroke mRS = 3). Secondary endpoints were the rate of successful target vessel recanalization and complication rate including intracranial hemorrhage in follow-up imaging. Exclusion criteria were a severe stroke syndrome (NIHSS > 20) or decreased level of consciousness, intubation before transport to stroke center, simultaneous LVO of the anterior circulation, or re-stroke within 3 months of follow-up. The two study groups were defined as patients with LVO of the posterior circulation treated under CS compared to patients with LVO of the posterior circulation treated under elective GA.

### Performance of Modern EST for LVO in the Posterior Circulation

Decision for EST was made based on a consensus by the neurologist and neurointerventionalist after initial stroke imaging with CT or MRI. Intravenous thrombolysis was administered according to national and international guidelines. The choice of the sedation mode in the complete study cohort was made according to the patient's compliance, severity of the stroke syndrome, and level of consciousness. The bias of selecting more severely harmed patients preferably for general anesthesia was reduced in this study by the abovementioned exclusion criteria (severe stroke syndrome, decreased level of consciousness, intubation before transport).

In the standard approach for EST, a transfemoral access is conducted followed by placing a guide catheter in the subclavian artery (7F/80 cm Flexor Shuttle, Cook Medical, Bloomington, IN, USA). Subsequently, a distal access catheter is introduced to the vertebral artery (e.g., Sofia 5F, MicroVention, Aliso Viejo, CA, USA). The first-line approach (performing direct thromboaspiration or stent-retriever-thrombectomy in combination with continuous distal aspiration using a distal aspiration catheter), as well as the choice of material used for EST, is at the discretion of the treating neurointerventionalist. The following stent-retriever types were used (descending order of frequency of use): Solitaire (Medtronic, Dublin, Ireland), Trevo (Kalamazoo, MI, USA), pRESET (phenox, Bochum, Germany). Follow-up imaging is performed 24 h after EST with either CT or MRI.

### Anesthesia Protocols During EST (CS and GA)

Peri-interventional management in both groups was conducted entirely by neurointensivists trained in the interventional setting and a specialized neurocritical care nursing team, according to standard operating procedures derived from guideline recommendations and established over years and continuously adapted to local conditions (2, 13, 14).

### Data Acquisition

Source data were generated from a prospectively collected stroke database. Additionally, all data included in the present analysis were validated retrospectively to minimize incorrect or missing data (CW, MM). The pre- and post-interventional stroke imaging was reviewed to assess the posterior circulation Alberta Stroke Program Early CT Score (pc-ASPECTS) and post-interventional intra-cranial bleeding.

### Statistical Analysis

Data are shown as median with interquartile range (IQR) or means with standard deviation (SD), as appropriate. Normal distribution was tested for each variable using the Shapiro–Wilk test. Two-sided *t* tests, χ^2^ test, or Mann–Whitney U test was performed as appropriate to compare groups. The level of significance was set at.05 for all *p*-values. The statistical analyses were performed by using software (SPSS Statistics 21.0.0.0; IBM, Armonk, NY). For the outcome analysis, the groups were compared in univariate and 1:1 matched-pair analysis without propensity score or other applied statistical models (15). Matching criteria were neurological deficit on admission (matching NIHSS ± 4 points), matching pre-stroke mRS, and reperfusion result based on the modified Treatment in Cerebral Ischemia (mTICI) Classification.

## Results

**Figure 1:**
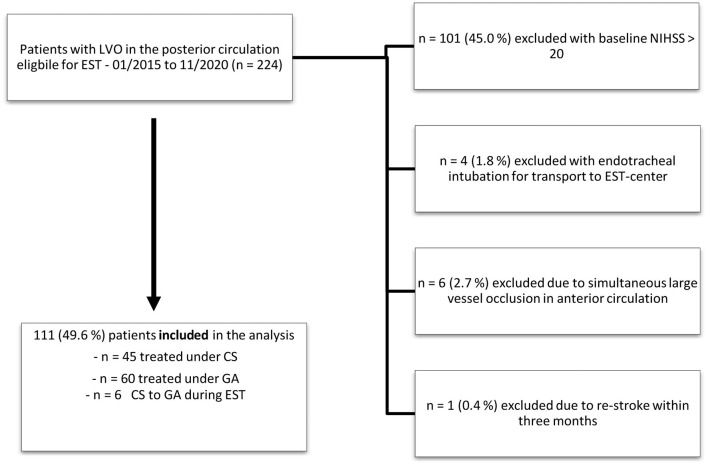
Patient selection for comparison of patients with stroke in the posterior circulation according to the applied mode of anesthesia. EST, endovascular stroke treatment; NIHSS, National Institute of Health Stroke Scale; LVO, large vessel occlusion.

Of the 224 patients treated with EST for acute ischemic stroke in the posterior circulation between January 2015 and November 2020, 101 were excluded due to severe stroke syndromes (NIHSS > 20) and consequently mandatory GA. Of the remaining 123 patients, 11 patients were excluded due to simultaneous LVO in the anterior circulation (*n* = 6), endotracheal intubation before transportation to our facility (*n* = 4), or re-stroke within 3 months (*n* = 1). In six included cases, the sedation mode was changed from CS to GA during MT. In four cases, the change in sedation mode was performed to guarantee patient immobility for stent-assisted PTA of the basilar artery. In two cases, GA was required due to patient agitation—see Figure 1.

### Outcome Analysis

In this study, univariate analysis showed that patients treated under CS were less likely to be male [*n* (%): 21 (46.7) CS vs. 41 (68.3) GA, *p* = 0.025] and showed more often a basilar artery tip or posterior artery occlusion as target vessel occlusion [23 (51.1) CS vs. 17 (28.3) GA, *p* = 0.017]. They also had a higher baseline pc-ASPECTS (10 (8–10) CS vs. 9 (7–10) GA, *p* = 0.023), had a shorter time interval from stroke imaging to groin puncture [45 (31–62) CS vs. 64 (45–100) GA, *p* = 0.004], and required less intracranial stenting due to underlying atherosclerotic stenosis of the target vessel [*n* (%): 3 (6.7) vs. 13 (21.7), *p* = 0.034]. While the baseline mRS (0 (0–1) CS vs. 0 (0–2) GA, p = 0.649) as well as the NIHSS on admission [7 (4–12) CS vs. 8 (5–14), *p* = 0.209)] were comparable between the two groups, in the matched-pair outcome analysis, patients treated under CS showed a lower mRS 90 days after stroke onset [2.5 (1–4) CS vs. 4 (2–6), *p* = 0.008] and a comparable rate of good functional outcome [*n* (%): 19 (42.2) CS vs. 18 (30.0) GA, *p* = 0.143].

Patient data are presented in Table 1.

**Table 1:** Group comparison of patients treated for LVO of the posterior circulation with EST under conscious sedation (CS) or general anesthesia (GA).

	**CS**	**GA**	***P*-value**
	**(*n* = 45)**	**(*n* = 60)**	
**Patient characteristics**			
Age, median (IQR)	75 (66–82)	76 (64–81)	0.933
Sex—male, *n* (%)	21 (46.7)	41 (68.3)	**0.025**
Diabetes mellitus, *n* (%)	8 (17.8)	9 (15.0)	0.789
Blood glucose level on admission, median (IQR) [mg/dl]	123 (107–150)	119 (104–144)	0.924
Hypertension	36 (80.0)	45 (75.0)	0.896
Atrial fibrillation	16 (35.6)	12 (20.0)	0.093
Hypercholesterinemia	14 (31.1)	17 (28.3)	0.935
Pre-stroke mRS, median (IQR)	0 (0–1)	0 (0–2)	0.649
NIHSS on admission, median (IQR)	7 (4–12)	8 (5–14)	0.209
**Time windows**			
Unknown symptom onset (“wake up stroke”) *n* (%)	15 (33.3)	24 (40.0)	0.484
Transfer for treatment *n* (%)	21 (46.7)	31 (51.7)	0.612
Intra-venous thrombolysis *n* (%)	16 (35.6)	23 (38.3)	0.856
Time from onset to imaging, median (IQR) [min]	142 (62–557)	228 (79–622)	0.084
Time from imaging to groin puncture, median (IQR) [min]	45 (31–62)	64 (45 – 100)	**0.004**
Time from groin puncture to final EST-result, median (IQR) [min]	55 (32–88)	68 (47–123)	0.177
**Imaging and Interventional characteristics of EST**			
Basilar artery occlusion, *n* (%)	20 (44.4)	49 (81.7)	** <0.001**
Tip of the basilar artery/posterior artery, *n* (%)	23 (51.1)	17 (28.3)	**0.017**
Dominant V4 segment of vertebral artery, *n* (%)	2 (4.4)	4 (6.7)	0.631
Intra-cranial stenting of target vessel	3 (6.7)	13 (21.7)	**0.034**
ADAPT as first thrombectomy attempt, *n* (%)	9 (20.0)	10 (16.7)	0.931
Thrombectomy attempts in total, median (IQR)	1 (1–2)	1 (1–2)	0.753
Successful revascularization (mTICI 2–3), *n* (%)	38 (84.4)	51 (85)	0.762
Failure of revascularization (mTICI 0–1), *n* (%)	2 (4.4)	5 (8.3)	0.501
**Procedural complications**			
Intraprocedural complications	3 (6.7)	5 (8.3)	0.743
Intracranial vasospasms, *n* (%)	1	1	
Vessel perforation, *n* (%)	0	0	
Vessel dissection, *n* (%)	0	2	
Thrombus embolization, *n* (%)	2	2	
**Follow-up imaging**			
pc-ASPECTS baseline, median (IQR)	10 (8–10)	9 (7–10)	**0.023**
pc-ASPECTS follow-up, median (IQR)	8 (7–10)	7 (5–9)	**0.012**
Intracranial hemorrhage, *n* (%)	4 (8.9)	7 (11.7)	0.705
Symptomatic intracranial hemorrhage	2	3	0.586
**Clinical outcome (matched-pair analysis)**			
mRS discharge, median (IQR)	3 (2–4)	4 (3–5)	**0.005**
NIHSS discharge, median (IQR)	2.5 (1–11)	6.5 (2–23)	**0.034**
mRS 90 days after stroke onset, median (IQR)	2.5 (1–4)	3 (2–6)	**0.036**
Good outcome (mRS 0–2 or 3 if pre-stroke mRS 3) 90 days after stroke onset, *n* (%)	19 (42.2)	15 (32.6)	0.311
Mortality 90 days after stroke onset, *n* (%)	4 (8.9)	14 (31.1)	**0.002**

*Bold values are statistically significant p-values (<0.05)*.

### Recanalization Result and Procedural Complications

The two study groups showed no difference in successful target vessel recanalization [*n* (%): 38 (84.4) CS vs. 51 (85.0) GA, *p* = 0.762] or failure of recanalization [*n* (%): 2 (4.4) CS vs. 5 (8.3) GA]. With an overall low complication rate in this study, there was no difference in complication rates between the groups [3 (6.7) CS vs. 5 (8.3) GA, *p* = 0.501]. The total number of thrombectomy attempts was alike [median (IQR): 1 (1, 2) CS vs. 1 (1, 2) GA, *p* = 0.753] as well as the rate of ADAPT technique for first recanalization attempt [9 (20.0) CS vs. 10 (16.7) GA, *p* = 0.931]. The study groups also did not differ regarding intracranial hemorrhage in follow-up imaging [4 (8.9) CS vs. 7 (11.7) GA, *p* = 0.705].

## Discussion

The optimal mode of anesthesia in EST is still under debate. While retrospective analyses rather showed a benefit for CS, three randomized controlled trials addressing EST of the anterior circulation did not find a difference but rather a benefit in favor of GA (5). In contrast, for patients with acute ischemic stroke in the posterior circulation, evidence for the best choice of sedation mode during EST is sparse.

In this study, for the majority of patients (85%) a successful recanalization of the target vessel occlusion could be reached and the technical success rate is comparable to earlier studies on EST in the PC as well as to studies concerning EST in the anterior circulation (9). The success rate does not differ depending on the sedation mode during EST in this study cohort. Also, the complication rate is low for both study groups (6.7% CS vs. 8.3% GA). While the matched-pair outcome analysis is supposed to reduce the bias of treating clinically more affected patients in general anesthesia, the functional outcome is better for patients treated under CS. However, the study groups differ concerning baseline pc-ASPECTS, requirement of intracranial stenting, and exact location of target vessel occlusion with more basilar artery occlusions in the GA group. Thus, the presumably better functional outcome of patients treated under CS has to be seen under the light of the potential bias to treat more complicated cases under elective GA. Nevertheless, our study allows the conclusion, that EST under CS for eligible patients is time saving as well as safe and effective and thereby challenges the concept currently active in many stroke centers of intubating every patient with large vessel occlusion in the posterior circulation before EST.

There are only a few studies addressing the sedation mode during EST of the posterior circulation so far. Smaller observational studies from 2014 and before concentrated mainly on EST of the anterior circulation including patients with acute ischemic stroke of the posterior circulation to a varying degree. Precise conclusions for the posterior circulation could not be drawn (16, 17). Two larger-sized retrospective studies concentrating on sedation mode for EST in the posterior circulation showed differing results. Bouslama et al. dismissed CS during EST as an independent predictor for a favorable outcome in a retrospective study of *n* = 214 patients (18). Jadhav et al. showed comparable outcomes for EST under CS vs. GA, when analyzing *n* = 63 patients with vertebrobasilar occlusions treated under CS matched for admission glucose and NIHSS with patients treated under GA (19). These studies did not exclude patients intubated for transport, do not offer information about the rate of intracranial stenting in the study population, and included patients from early EST times before the third-generation stent retrievers were developed. With addressing the above-named shortcomings of predecessor studies, our study supports the results of Jadhav et al., that CS for EST in the PC is safe and effective.

The conversion rate from CS to GA during EST with *n* = 6/111 (5.4%) is comparable to studies of the anterior circulation, which showed a conversion rate of 6.3 to 11.5% (3, 5). Conversion from CS to GA was mainly conducted, when an intracranial stenting became necessary. Intracranial stenting under conscious sedation in the posterior circulation was very rarely performed in this study (in three patients). Jadhav et al. included *n* = 16 patients with intracranial stenting under CS. Generally, it is described that intracranial stenting under CS is feasible (20). However, there is still a relative lack of evidence for EST of the posterior circulation overall and especially regarding intracranial stenting in consciously sedated patients (9), a fact that contributes to differing therapy protocols in different stroke centers.

## Limitations

Limitations of this study are mainly related to the single-center retrospective design. Most likely, patients with complex vascular findings were treated under GA affecting the outcome analysis. The authors of this study want to state that they are well aware of the heterogeneity seen in this study cohort, which comes with analyzing posterior circulation ischemic stroke patients, where many stroke concepts of the anterior circulation (e.g., the penumbra concept) cannot be applied. Nevertheless, these study results show the feasibility of conscious sedation for treating eligible stroke patients and invite to develop better standard operating procedures for posterior circulation stroke patients. To achieve this, more studies addressing differences of stroke in the posterior circulation are needed. This study could serve as basis for prospective, randomized clinical trials and shows aspects, which need further investigation like sedation mode during intracranial stenting in EST of the posterior circulation.

## Conclusion

Performing endovascular stroke treatment of the posterior circulation under conscious sedation was safe and effective for eligible patients in this study. Compared to patients treated under general anesthesia, the rate of successful target vessel recanalization as well as the complication rate was alike. The clinical outcome comparison tends to favor treatment under conscious sedation, which reflects more likely a basic group difference of the two study groups than a veritable effect.

## Data Availability Statement

The raw data supporting the conclusions of this article will be made available by the authors, without undue reservation.

## Ethics Statement

The studies involving human participants were reviewed and approved by Ethikkommission der Medizinischen Fakultät Universitätsklinikum Heidelberg. Written informed consent for participation was not required for this study in accordance with the national legislation and the institutional requirements.

## Author Contributions

CW substantially contributed to the manuscript's concept development, data acquisition, statistical analysis and writing the manuscript. MC, LJ, and SS substantially contributed to data acquisition and manuscript revision. AP and FS substantially contributed to data acquisition, statistical analysis and manuscript revision. MB and MM substantially contributed to the manuscript's concept development and manuscript revision. All authors contributed to the article and approved the submitted version.

## Funding

We acknowledge financial support by the Open Access Publishing Fund of Ruprecht-Karls-Universität Heidelberg.

## Conflict of Interest

The authors declare that the research was conducted in the absence of any commercial or financial relationships that could be construed as a potential conflict of interest.

## Publisher's Note

All claims expressed in this article are solely those of the authors and do not necessarily represent those of their affiliated organizations, or those of the publisher, the editors and the reviewers. Any product that may be evaluated in this article, or claim that may be made by its manufacturer, is not guaranteed or endorsed by the publisher.

## Abbreviations

CS: conscious sedation
EST: endovascular stroke treatment
GA: general anesthesia
ICH: intracranial hemorrhage
LVO: large vessel occlusion
mTICI: modified treatment in cerebral ischemia
pc-ASCPETS: posterior circulation alberta stroke program early CT score.
